# Pre-existing weakness is critical for the occurrence of postoperative cognitive dysfunction in mice of the same age

**DOI:** 10.1371/journal.pone.0182471

**Published:** 2017-08-07

**Authors:** Yujie Tang, Xueqin Wang, Shuibing Zhang, Shangchun Duan, Wenxiang Qing, Gong Chen, Feng Ye, Yuan Le, Wen Ouyang

**Affiliations:** 1 Department of Anesthesiology, the Third Xiangya Hospital, Central South University, Changsha, Hunan, P.R. China; 2 Center for Experimental Medicine, the Third Xiangya Hospital, Central South University, Changsha, Hunan, P.R. China; 3 Key Laboratory of Molecular Biology for Infectious Diseases, Ministry of Education of China, The Second Affiliated Hospital, Chongqing Medical University, Chongqing, P.R. China; University of Virginia, UNITED STATES

## Abstract

Occurrence of postoperative cognitive dysfunction (POCD) is age-dependent and heterogenous. Factors deciding the occurrence of POCD in patients of the same age undergone same surgeries remain unclear. Here we investigated the effects of pre-existing weakness on the occurrence of POCD in mice of the same age. Pre-existing weakness of mice was induced by intraperitoneal injection of lipopolysaccharide (8mg/kg) and was evaluated by physical frailty index (by open field test), neuroinflammation level (by Iba1 immunostaining and inflammatory factors TNF-α and IL-1β), and neuronal activity (by p-CREB immunostaining). POCD was induced by partial hepatolobectomy and was evaluated by puzzle box test and Morris water maze test. The brains were collected to detect the levels of neuroinflammation, synaptophysin and NMDA receptor subunits NR2A, NR2B and NR1 (by western blot), and oxidative stress (by Dihydroethidium). Compared to the normal adult mice of the same age, LPS pretreated mice had increased physical frailty index, higher levels of neuroinflammation, and lower neuronal activity. Partial hepatolobectomy induced obvious impairments in executive function, learning and memory in LPS pretreated mice after surgery, but not in normal mice of the same age. Partial hepatolobectomy also induced heightened neuroinflammation, obvious loss of NMDA receptor subunits, strong oxidative stress in LPS pretreated mice on the 1^st^ and 3^rd^ postoperative day. However, the POCD-associated pathological changes didn’t occur in normal mice of the same age after surgery. These results suggest that pre-existing weakness is critical for the occurrence of POCD in mice of the same age.

## Introduction

Postoperative cognitive dysfunction (POCD), a common postoperative complication in the elderly patients, is characterized by subtle deficits in one or more discrete domains of cognition, e.g. attention, concentration, executive function, verbal memory, visuospatial abstraction and psychomotor speed [1,2]. It occurs in 30% to 80% of elderly patients during the first week after surgery, and 10% to 15% of elderly patients three month after surgery[3,4]. The occurrence of POCD is significantly associated with increased postoperative morbidity and mortality, greater length of hospital stay, increased costs, and decreased life independence[5]. Therefore, POCD has become a hot topic in biomedical research across the globe. Lots of studies have been done to investigate its risk factors, pathological mechanisms, and methods of prevention and treatment[2]. So far, neuroinflammation[6,7,8], oxidative stress[9,10] and synaptic changes[11,12] all have been reported to contribute to the development of POCD. Age is described as the only risk of long-term POCD[4]. However, factors deciding the occurrence of POCD in patients of the same age and undergone same surgeries remain unclear.

Previous studies have shown that POCD’s occurrence is age-dependent and heterogenous. For example, Monk et al categorized patients as young, middle-aged, and elderly group. Approximately 5.7% young, 5.6% middle-aged patients exhibited POCD at 3 month after surgery, while up to 12.7% elderly patients suffered from POCD at the same time[13]. Laalou et al detected that cognitive impairment was present in 23% patients between 60 and 69 years old 1 week after surgery, while the incidence of POCD was 29% in patients older than 70 years old[14]. Similar age-dependent phenomenon was also observed in young adult and aged animals. For example, Xu found that surgery impaired the cognitive function of rats aged eighteen month, but didn’t impair that of rats in nine-month-old. Further mechanism studies showed that peripheral surgery increased the levels of A-beta 40 and A-beta 42 in the hippocampus of aged rats, but didn’t increase the levels of young adult rats[15]. Le’s research found that peripheral surgery decreased the spine density of aged rats, but had no obvious effects in adult rats[16]. Further studies have shown that, for elderly patients of the same age and undergone same surgeries, POCD more commonly occurred in patients who exhibited preoperative co-morbidities, e.g. pre-existing cognitive impairment[17], decreased hippocampal volume[18], reduced blood flow in the left middle cerebral artery[19], etc. Thus, we propose that pre-existing weakness is crucial for the occurrence of POCD in mice of the same age.

Septic survivors usually have physical, cognitive, and mental impairments[20,21,22], which significantly increased individual weakness. In this research, we induced pre-existing weakness of mice by intraperitoneal injection of lipopolysaccharide (8mg/kg)[23]. Partial hepatolobectomy was used to induce POCD[16]. Our data showed that pre-existing weakness obviously affected the occurrence of POCD and the related pathological changes.

## Materials and methods

### Ethics statement

Experiments were performed in accordance with the guidelines for experimental animal use of the Central South University. The protocol (LLSC (LA).2015–016) was approved by the ethics committee of the 3^rd^ Xiangya Hospital of Central South University.

### Animals and experiments

C57BL6J male mice (2 months, 20-25g) were purchased from Central South University (P.R. China). All animals were maintained at 23±3℃ on a 12-h light and 12-h dark cycle with free access to food and water. Those mice were adapted to their environment for 7 days before the experiments.

### Experiment 1

In order to identify whether LPS pretreated mice exhibit higher level of physical frailty index and brain vulnerability than normal mice, mice and LPS pretreated mice were used to detect the movement in open field test (n = 12 per group)[24], the level of neuroinflammation (n = 3 per group for RT-PCR, n = 4 per group for Iba1 immunostaining)[6,7,8], and neuronal activity (n = 3 per group for p-CREB immunostaining)[25,26]. LPS pretreated mice were induced via intraperitoneal injection of 8 mg/kg LPS (Lipopolysaccharides, Sigma-Aldrich, USA, catalog number: L2880) into the adult mice and survived for five days[27], while normal mice received saline. For mechanism exploration, mice were sacrificed on the fifth day.

During the first 48h after intraperitoneal injection, all mice were immediately placed to warm environment and received subcutaneous injection of warm saline(0.5ml) every 8 hours for fluid resuscitation. Softened food was used to fed. All measures were taken to relieve the suffering. The survival of all mice who received LPS was monitored for 4 days before the hepatolobectomy. During the first 48 hours, mice were monitored every 6 hours, and then once daily until the surgical day. The criteria used for humane endpoints were as follows, hunched posture, decreased activity, labored breathing, loss of appetite and piloerection[28]. Experiments were indeed stopped if mice met the above criteria. They were euthanized with sevoflurane, and death was verified by monitoring cardiopulmonary arrest. There was no death after receiving LPS.

### Experiment 2

In order to verify whether mice with pre-existing weakness was associated with exaggerated postoperative cognitive dysfunction, adult mice were divided into two parts. One part was used as normal adult mice. The other part was intraperitoneally injected 8 mg/kg LPS and used as LPS pretreated mice. Five days later, two parts of mice were divided into control and surgery groups. Mice in the surgery group received anesthesia and partial hepatolobectomy. In contrast, mice in control group didn’t receive anesthesia and partial hepatolobectomy. The behavioral test was begun on the 2^nd^ postoperative day (n = 11 in Normal; n = 12 in Normal + S/ LPS pretreated; n = 13 in LPS pretreated + S). The mice used for mechanism exploration were sacrificed on the 1^st^ and 3^rd^ postoperative day (n = 5 per group for Iba1 immunostaining; n = 3 per group for RT-PCR, ROS detection; n = 4 per group in control part, n = 3 per group in surgery part for western blot).

### Anesthesia and partial hepatolobectomy

Anesthesia was prepared with the procedure described by He et al[29]. Briefly, mice were rapidly anaesthetized by inhalation of 3% sevoflurane with high flow of oxygen (5L/min) using a mask. The anesthesia was maintained with continuous delivery of 2% sevoflurane with oxygen (80% -85%). The gas was monitored and analyzed by a multi-function monitor (Datex-Ohmeda, Helsinki, Finland). Respiratory rate, ETCO2, FiO2 and FiSev were also monitored. The depth of anesthesia was modulated according to the respiratory rate (30-50/min) and the body movement. The anesthesia duration for each mouse was 2 hours.

The partial hepatolobectomy was performed as previously described [30]. Briefly, a transverse incision about 2 cm long was made below the xyphoid; the left lobe of liver was carefully isolated, ligated, and then removed. Finally, muscles and skin were closed with sterile sutures. 0.25% bupivacaine(50ul) [31] was injected subcutaneously for the purpose of local postoperative analgesia. After received anesthesia and hepatolobectomy, mice were placed to warm place and observed until complete awakening. The survival of all mice who undergone surgery was monitored for 3 days consecutively. During the first 24 hours, mice were monitored every 6 hours, and then once daily. If mice meet the criteria used for humane endpoints, they would be euthanized with sevoflurane as mentioned above. Although all efforts were made to minimize suffering, there were four mice in LPS pretreated + surgery group died under general anesthesia unexpectedly.

### Open field test

Locomotor activity was tested in the open field as described in a previous study[12]. Briefly, the mouse was placed directly into the center of the open field(50×50cm), Movement of the animal in the area was recorded by computerized video track system (Logitech, Suzhou, China). The total distance traveled and mean speed were analyzed by smart junior software 3.0 (Panlab, Cambridge, USA).

### Frailty index

The mouse’s physical frailty index was evaluated according to the mouse’s movement in the open field and weight following the reported method. The method was following Jocelyne C et al and made little adjustment [24]. Briefly, mice were weighed before testing. Activity of mice in an open-field arena (50 ×50cm) were recorded with computerized video track system (Logitech, Suzhou, China) for 10 minutes. Videos analyzed with smart junior 3.0 software (Panlab, Cambridge, USA) and manual work to obtain the following seven parameters: (a) total distance moved in 10 minutes(cm); (b) maximal distance moved between bouts of inactivity (cm); (c) total duration of movement (seconds); (d) percent of total time spent moving; (e) the maximum speed(cm/s); (f) the average velocity of movement over 10 minutes (cm/s); and (g) rearing frequency (number of occurrences/10 min). According to these eight parameters, we calculated the mean value and SD value in normal mice group and used as reference values. Individual values for each parameter measured in each mouse were compared with reference values. Values that were 1 SD above or below the mean reference valued were given a score of 0. Values that were ±1 SD differed from the mean reference value were given a frailty value of 0.25. Those that differed by ±2 SD were scored as 0.5, values that differed by ±3 SD were given a value of 0.75, and values that were more than 3 SD above or below the mean received the maximal frailty value of 1. These values were summed, and then divided by the total number of items measured. The frailty index score of each animal ranged from 0 to 1.

### Puzzle box test

Executive function was assessed following a protocol that was similar to those described in previous studies [32]. A puzzle box which consisted of a start box and a goal box were used in this test. A doorway and an underpass connected the start box with the goal box, which allowed the animals to move between compartments. Mice were tested over a 3-day period. To reach the goal (darkened) box, mice need to overcome progressively more difficult tasks. On day 1, trial 1, the doorway and underpass was open(baseline). On day 1, trial 2, the doorway was closed and the underpass was open (problem solving). Trial 3 was conducted 2 min later and repeated trail 2 (short-term memory for underpass task). Trials 4–6 were conducted on day 2. The trial 4 was the same as trail 2 and trail 3(long-term memory for underpass task). During trial 5 the task became more difficult as the doorway was closed and the underpass was covered with corncob bedding, the mouse needs to burrow through the bedding to arrive at the goal box (problem solving). Trial 6 was repeated 2 min after trial 5 (short-term memory for burrowing task). Trial 7 were conducted on day 3. During trial 7, the doorway was closed and the underpass was obstructed with corncob bedding (Long-term memory for burrowing task). After reached the goal box, the mouse was allowed to stay in the goal box for 2 min, Short-term memory was assessed 2 min after first exposure to the task. Long-term memory was assessed 24 hours after first exposure to the task. The maximum trial time was 5 min for a trial. The time for each animal to access the goal box was recorded.

### Morris water maze test

The Morris water maze test was begun on the fifth day after surgery to assess the learning and memory of mice. We used a computerized video track system (Logitech, Suzhou, China) to record the mice’s movement in water maze by following our previous method[29]. Briefly, a transparent round platform was placed 1 cm below the water surface of southeast quadrant in a circular pool. During the training, mice were first placed on the platform for 30 seconds, and then were released into the water facing the tank wall. During each trial, a maximum time of 60 seconds was allowed for the mouse to find the platform. If a mouse couldn’t locate the platform within 60 seconds, it was guided to the platform and remained for 30 seconds. All mice were trained for 4 days with three trials per day, and the latency to platform during each trial was recorded. Four days after training, the platform was taken out of the pool. Mice were released into the water facing the tank wall from the northwest quadrant. The movements of mice within 60 seconds were recorded. And the memory of mice was evaluated by the latency for the first entrance of targeted area and the percentages of searching time in the targeted area.

### Brain cytokine mRNA measurement by quantitative real-time PCR

Total RNA was extracted from homogenized hippocampal tissues using Trizol Reagent (Invitrogen, USA, catalog number: CA02008). According to manufacturer’s instruction, cDNA was synthesized using All-in-One^TM^ First-Strand cDNA Synthesis Kit (GeneCopoeia, USA, catalog number: AORT-0020). Quantitative real-time PCR was performed using All-in-One^TM^ qPCR Kit (GeneCopoeia, USA, catalog number: QP001). Gene-specific primers, forward and reverse, were as follows: 5′- ATGCACCACCATCAAGGACTCAA -3′ and 5′- ACCACTCTCCCTTTGCAGAACTC-3′ for TNF-α, 5′- GCCCATCCTCTGTGACTCAT-3′ and 5′-AGGCCACAGGTATTTTGTCG-3′ for IL-1β, 5′-GGTGAAGGTCGGTGTGAACG-3′ and 5′-CTCGCTCCTGGAAGATGGTG-3′ for GAPDH. Quantitative real-time PCR was performed at 95°C/10 min and then cycling 95°C/10 s, 60°C /20 s, 72°C/20 s, terminated by heating to 72°C/5 min. The products were amplified for 40 cycles. The relative expression level of mRNA was normalized to that of GAPDH and was finally calculated utilizing the 2(-Delta Delta C(T)) method[33].

### Western blot

Western blotting was used to assess the expression of NR2A, NR2B, NR1, synaptophysin, and GAPDH in the hippocampus. Briefly, frozen hippocampus was homogenized in a lysis buffer containing protease inhibitors cocktails (Roche, Germany, catalog number: P8340). The quantity of protein of samples was determined using a BCA protein assay kit (CWbio, China, catalog number: CW0014) according to the manufacturer’s instructions. Equal amounts of protein samples (/lane) were separated by sodium dodecyl sulfate polyacrylamide gel electrophoresis (SDS-PAGE) and transferred to polyvinylidene fluoride membranes. After washing, membranes were blocked with 10% skim milk in TBST buffer for 1 h and then incubated with primary antibodies (rabbit polyclonal antibody to NR2A: 1:1000, Abcam, USA, catalog number: ab169873; rabbit polyclonal antibody to NR2B: 1:2000, Proteintech, USA, catalog number: 21920-1-Ap; Rabbit monoclonal antibody to NMDAR: 1:1000, Cell Signaling Technology, USA, catalog number: 5704; rabbit polyclonal antibody to synaptophysin: 1:2000, Proteintech, USA, catalog number: 17785-1-1-AP; rabbit polyclonal antibody to GAPDH: 1:2000, Proteintech, USA, catalog number: 10494-1-AP) overnight at 4°C. After three times washing, membranes were incubated with the secondary antibody (1:2000, CWbio, China, catalog number: CW0160S) at room temperature for 2h. Finally, protein was visualized by enhanced chemiluminescence detection kit (CWbio, China, catalog number: CW0049), and the intensity of each band was quantified by densitometry. Relative expression levels of protein were normalized by the ratio of targeted protein (NR2A, NR2B, NR1, synaptophysin) to GAPDH.

### Immunostaining

Mice were anesthetized with sevoflurane and perfused transcardially with 0.01 M phosphate-buffered saline (PBS). The brain was removed out. One half of the brain was used for immunostaining, the other half was used for western blot. The brain used for immunostaining was fixed in 4% paraformaldehyde overnight at 4°C. After dehydrating with sucrose, the brains were embedded in OCT, and coronal section of the brain (20 μm) were serially cut using a cryostat. According to the reported method of Liang et at[34], 5 sections of hippocampus (at levels of -1.22, -1.42, -1.62, -1.82, and -2.02mm relative to the bregma) were picked out for Iba1 and p-CREB immunostaining. After washing three times in 0.01 M PBS, these sections were blocked with 5% BSA in 0.01M PBS plus 0.1% Triton X-100 for one hour at room temperature and then incubated in primary antibody (rabbit polyclonal antibody to Iba1: 1:1500, Wako, Japan, catalog number: 019–19741; rabbit polyclonal antibody to p-CREB: 1:500, Abcam, USA, catalog number: ab32096) at 4°C overnight. After washing three times in 0.01M PBS, the sections were incubated in secondary antibodies (1:200) for two hour at room temperature and then washed three times. Finally, the sections were incubated in ABC (1:1:200) for one hour at room temperature. Followed by three times washes, a DAB kit was used to visualized.

For Iba1 immunostaining, two fields of cornu ammonis-1 (CA1) and five fields of dentate genus (DG) in all sections were captured. For p-CREB immunostaining, one field of CA1 and one field of dentate genus (DG) in all sections were captured. All pictures were taken with a microscope (Eclipse 80i, Nikon, Japan) under the same magnification (40x objective lens) and light intensity by an author who was blind to the experimental conditions. Based on the Iba1 staining, the percent of activated microglia in CA1 and DG was also counted by following the method reported by Cerbai et al[35]. According to the report, resting microglia was defined when the cell body was small and round, and branches were thin, highly ramified, equally distributed around the cell body. In contrast, activated microglia was defined when the cell body was bigger, pleomorphic bi- or tri-polar, or spindle, and branches were shortened, twisted or no ramification. In addition, the intensity of p-CREB in sections was analyzed by mean optical density. OD means integrated optical density (IOD) per stained area (μm^2^) (IOD/area) for positive staining. Image-Pro Plus 6.0 software was used[36].

### Reactive oxygen species detection

Similar to the reported method of Alberto et al[37], DHE (Dihydroethidium, Beyotime, China, catalog number: S0063) was used to detect reactive oxygen species production in the hippocampus of mice. Briefly, DHE was solubilized in 50% DMSO in 10mmol/L phosphate buffer. All animal groups received a series of two 9mg/kg intraperitoneal injections of DHE, separately by 1.5h. 24 hours after the initial injection, mice were anesthetized with sevoflurane and perfused transcardially with 0.01 M phosphate-buffered saline (PBS). In the next, the brains were immediately frozen in liquid nitrogen and then were embedded in OCT. Transverse sections of the brain (25 μm) were serially cut using a cryostat. These sections were quickly mounted on slides and covered with Dapi-Fluoromount-G (Vector laboratories, Burlingame, USA, catalog number: CA 94010), and then were observed with a microscope (Eclipse 80i, Nikon, Japan) under the same magnification (20x objective lens). Ethidium fluorescence (excitation at 300nm, emission at 610 nm) was examined and captured. CA1 and DG from 5 sections in the hippocampus (at levels of -1.22, -1.42, -1.62, -1.82, and -2.02mm relative to the bregma[34]) were analyzed per animal. The Ethidium fluorescence was quantified from the images by an investigator who was blind to the experimental conditions. Software Image-pro Plus 6.0 was used to analyze.

### Statistical analysis

Weakness related data were analyzed by unpaired Student’s t-test or one-way ANOVA with the post hoc Bonferroni test. The puzzle box and Morris water maze data were analyzed by repeated measures ANOVA with the treatment as between-subjects factor and measure time as with-in subject factor. Biochemical data were analyzed by two-way ANOVA with the post hoc Bonferroni test. All data were presented as mean ± standard error (mean ± SEM) and analyzed by using Prism 5 (Graph Pad Software Inc, La Jolla, CA, USA). p<0.05 was considered statistically significant.

## Results

### LPS pretreated mice exhibit higher physical frailty index and brain vulnerability than age-matched normal adult mice

**Fig 1:**
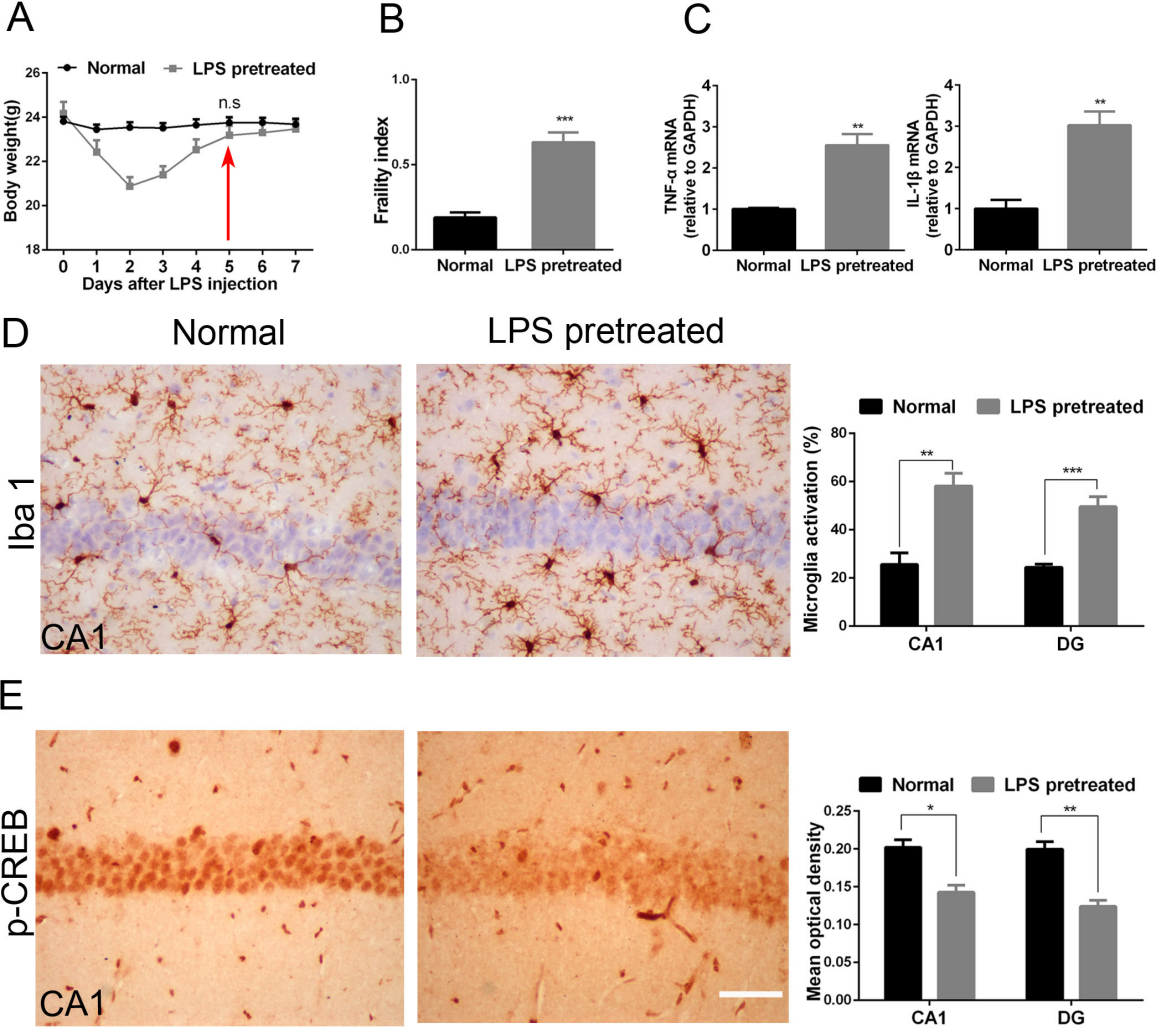
LPS pretreated mice exhibit higher physical frailty index and brain vulnerability than age-matched normal adult mice. (A) The trends of mice’s weight after received LPS intraperitoneal injection; (B)The performance-based eight-item frailty index. One-way ANOVA; Normal vs. LPS pretreated, p<0.001; n = 12 per group. (C) RT-PCR of TNF-α and IL-1β in the hippocampus. unpaired Student’s t-test; p = 0.004 for TNF-α; p = 0.007 for IL-1β; n = 3 per group. (D) Activated microglia of hippocampal CA1 and DG in normal mice and LPS pretreated mice. unpaired Student’s t-test; p = 0.002 in CA1; p<0.001 in DG; n = 4 per group (E) P-CREB expression in CA1 and DG of hippocampus in normal mice and LPS pretreated mice. unpaired Student’s t-test; p = 0.01 in CA1; p = 0.003 in DG; n = 3 per group. LPS pretreated mice correspond to 5 days survivors. All data were presented as mean±SEM; *p<0.05, **p<0.01, ***p<0.001. Bar = 50μm.

Weight is an important marker of health in mice. We used the weight recovery to define the disease recovery of LPS pretreated mice. Mice was intraperitoneally injected with 8 mg/kg LPS. The weight of LPS pretreated mice was similar to that of age-matched normal adult mice 5 days after LPS injection (23.76±0.24 vs 23.18±0.43, p = 0.23, Fig 1A). Hence LPS pretreated mice were used in the following experiments until the fifth day after LPS treatment.

Similar to elderly individuals, weak individuals were characterized by increased physical frailty index[24], structural and functional changes in brain including increased neuroinflammation, primed microglia[38] and low neuronal activity[25,26]. Interestingly, the physical frailty index of LPS pretreated was significantly higher than that of the age-matched normal adult mice (0.63±0.2 vs 0.18±0.1, p<0.001, Fig 1B). Compared to the normal adult mice, the mRNA levels of inflammatory factors TNF-α and IL-1β and the percentages of microglia activation in the hippocampus of LPS pretreated mice all increased obviously (p<0.05 for TNF-α, IL-1β, and percentages of microglia activation in CA1 and DG, respectively, Fig 1C and 1D). In addition, p-CREB is a common marker of neuronal activity, and its expression decreases in an age-dependent manner. Interestingly, p-CREB expression in the CA1 and DG of LPS pretreated mice was also obviously lower than that of normal adult mice (p = 0.01 in CA1, p = 0.003 in DG, Fig 1E). These results suggested that LPS pretreated mice recapitulated the features of weak individuals in physical frailty, neuroinflammation and neuronal activity.

### Hepatolobectomy induced obvious postoperative cognitive dysfunction in LPS pretreated mice, but not in age-matched normal adult mice

**Fig 2:**
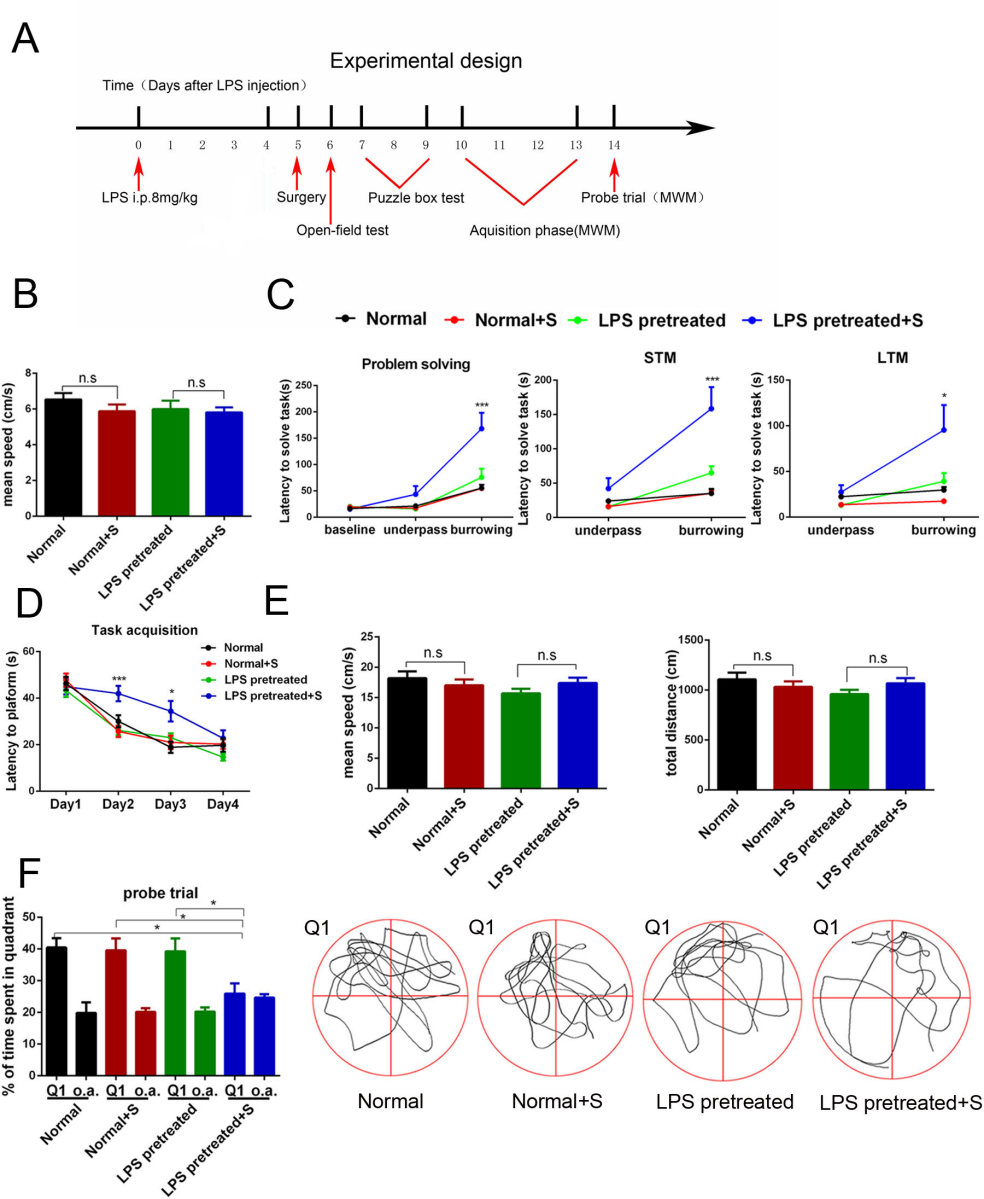
Hepatolobectomy induced obvious postoperative cognitive dysfunction in LPS pretreated mice, but not in age-matched normal adult mice. (A) Diagram of experimental design for the behavior test. (B) The movement speed of all groups was recorded one day after surgery. One-way ANOVA; p>0.05 (C) All mice spend similar latency when they go through the door or underpass to get to the destination on day1. For more difficult task on day 2, LPS pretreated plus surgery mice used longer time to burrow through bedding. In the later, it also exhibit significant impairment on short-term memory and long-term memory task. Two-way RM ANOVA; LPS pretreated mice vs. LPS pretreated mice + surgery; effect of surgery, p = 0.006; effect of measure time, p<0.001; effect of interaction, p = 0.003; p<0.001 for problem solving; p<0.001 for STM; p< 0.01 for LTM. (D) During the acquisition phase, on day 2 and day 3, surgery mice in LPS pretreated group exhibit longer latency to get to the target, while no significant difference was detected in normal surgery mice. Two-way RM ANOVA; LPS pretreated mice vs. LPS pretreated mice + surgery; effect of surgery, p = 0.003; effect of measure time, p<0.001; effect of interaction, p = 0.004; p<0.001 on day 2, p<0.05 on day 3. (E) Average swimming speed and total distance in probe trail phase was similar across groups. One-way ANOVA; p>0.05 respectively. (F) During the probe trail, percentages of time in the targeted area (Q1) of LPS pretreated plus surgery mice were less than the other three groups’ mice. One-way ANOVA; p<0.05. o.a. = average time spent in all other quadrants of the same group. All data were presented as mean±SEM; n = 11 in Normal; n = 12 in Normal + S/ LPS pretreated; n = 13 in LPS pretreated + S; *p<0.05, ** p<0.01, ***p<0.001.

The time schedule of the behavior tests was showed in Fig 2A. There was no obvious difference of movement speed among four groups in the open field on the first day after surgery (p = 0.54, Fig 2B). In the puzzle box test, hepatolobectomy didn’t exert obvious effects on the behaviors of normal adult mice and LPS pretreated mice at the learning and memory of easy task (Normal vs. Normal + surgery p = 0.99 through door, p = 0.99 through underpass, p = 0.97 for underpass STM; LPS pretreated mice vs. LPS pretreated mice + surgery, p = 0.99 through door, p = 0.35 through underpass, p = 0.53 for underpass STM, Fig 2C). But hepatolobectomy significantly increased the latency of LPS pretreated mice at the learning and memory of difficult burrowing task (p<0.001 for problem solving, p<0.001 for short-term memory, p = 0.004 for long-term memory, Fig 2C), suggesting the impairment of executive function and memory. During the training phase of Morris water maze test, hepatolobectomy significantly increased the latency for the first entrance of targeted area in LPS pretreated mice (p<0.001 on day 2, p = 0.03 on day 3, Fig 2D), but didn’t affect the latency of normal adult mice (p = 0.72 on day 2, p = 0.95 on day 3, Fig 2D). In the probe trial of Morris water maze test, hepatolobectomy decreased the time percent in targeted quadrant of LPS pretreated mice (p = 0.04 Fig 2F), while no significant difference was observed in normal adult mice (p = 0.9, Fig 2F). In addition, there was no difference detected in the mean speed and total distance of four groups during the probe trial (p = 0.31 for mean speed, p = 0.29 for total distance, Fig 2E). These results suggested that hepatolobectomy induced obvious impairments of executive function, learning and memory in LPS pretreated mice, but not in normal adult mice.

### Hepatolobectomy obviously aggravated neuroinflammation in the hippocampus of LPS pretreated mice, but not in age-matched normal adult mice

Neuroinflammation is an important pathological mechanism of POCD[6,7,8]. So we evaluated the postoperative neuroinflammation by detecting the percentages of microglia activation, and the expression levels of TNF-α and IL-1β mRNA.

**Fig 3:**
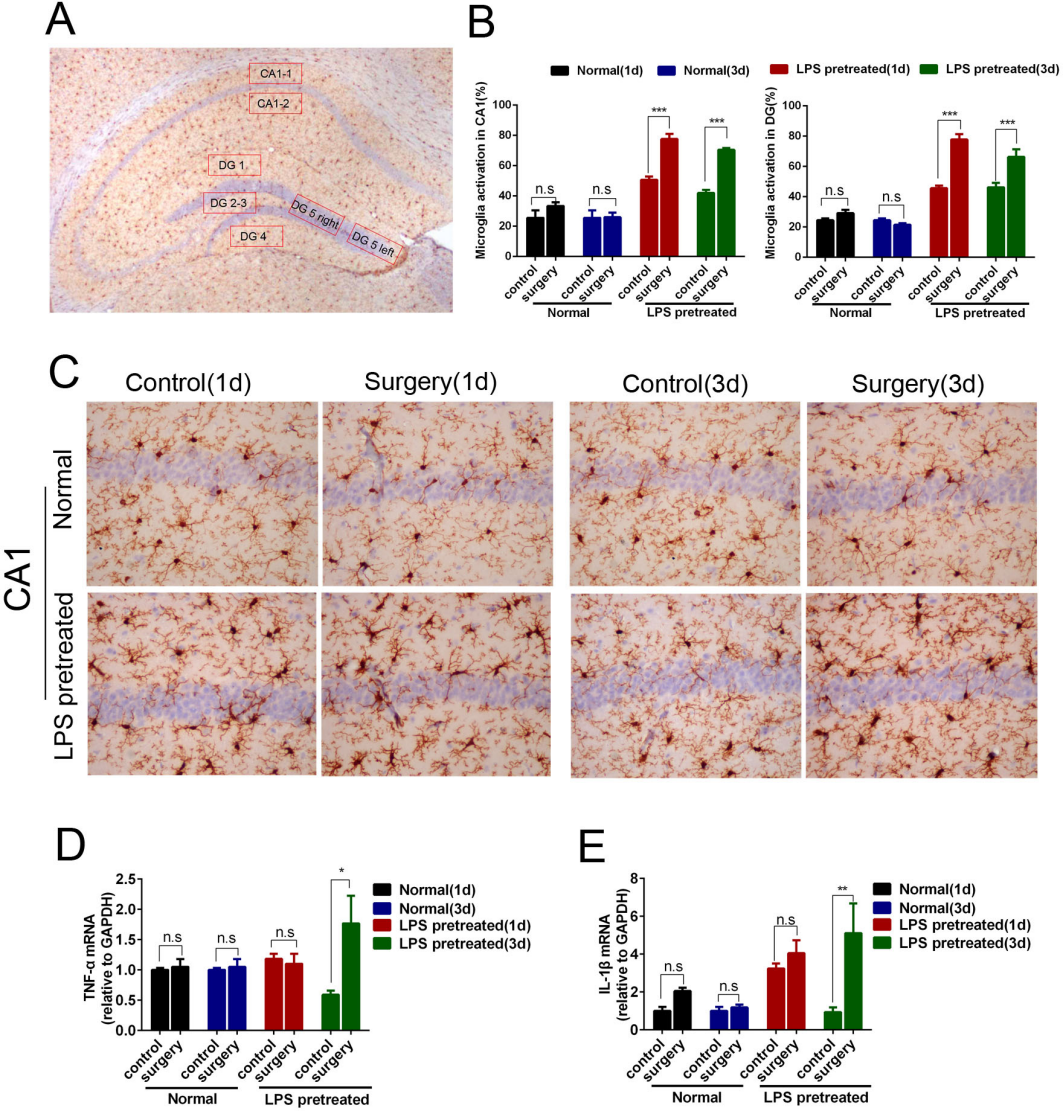
Hepatolobectomy obviously aggravated neuroinflammation in hippocampus of LPS pretreated mice, but not in age-matched normal adult mice. (A) Schematic diagram of 7 selected areas in hippocampal CA1 and DG. (B) Percentages of activated microglia in hippocampal CA1 and DG of all groups. Hepatolobectomy significantly increased the activated microglia of CA1, DG 1 d and 3 d after surgery in LPS pretreated mice. p<0.001 respectively, n = 5 per group. (C) Iba1 immunostaining of CA1 at hippocampus in normal mice and LPS pretreated mice. (D) RT-PCR of TNF-α and IL-1β in the hippocampus. p<0.05 for TNF-α; p<0.001 for IL-1β; n = 3 per group. LPS pretreated mice (1d) correspond to 6 days survivors, LPS pretreated mice (3d) correspond to 8 days survivors. All data were presented as mean±SEM and were analyzed by two-way ANOVA; *p<0.05, ** p<0.01, ***p<0.001. Bar = 50μm.

Hepatolobectomy increased the percentages of microglia activation in the hippocampal CA1 and DG of LPS pretreated mice 1 day and 3 days after surgery (p<0.001 respectively, Fig 3B and 3C). In addition, hepatolobectomy also up-regulated the mRNA levels of TNF-α and IL-1β (p = 0.02 for TNF-α; p<0.001 for IL-1β, respectively, Fig 3D). In contrast, hepatolobectomy didn’t induce such obvious changes in normal adult mice(p>0.05 respectively, Fig 3B–3D).

### Hepatolobectomy induced the selective loss of NMDA receptors in the hippocampus of LPS pretreated mice, but not in aged-match normal adult mice

**Fig 4:**
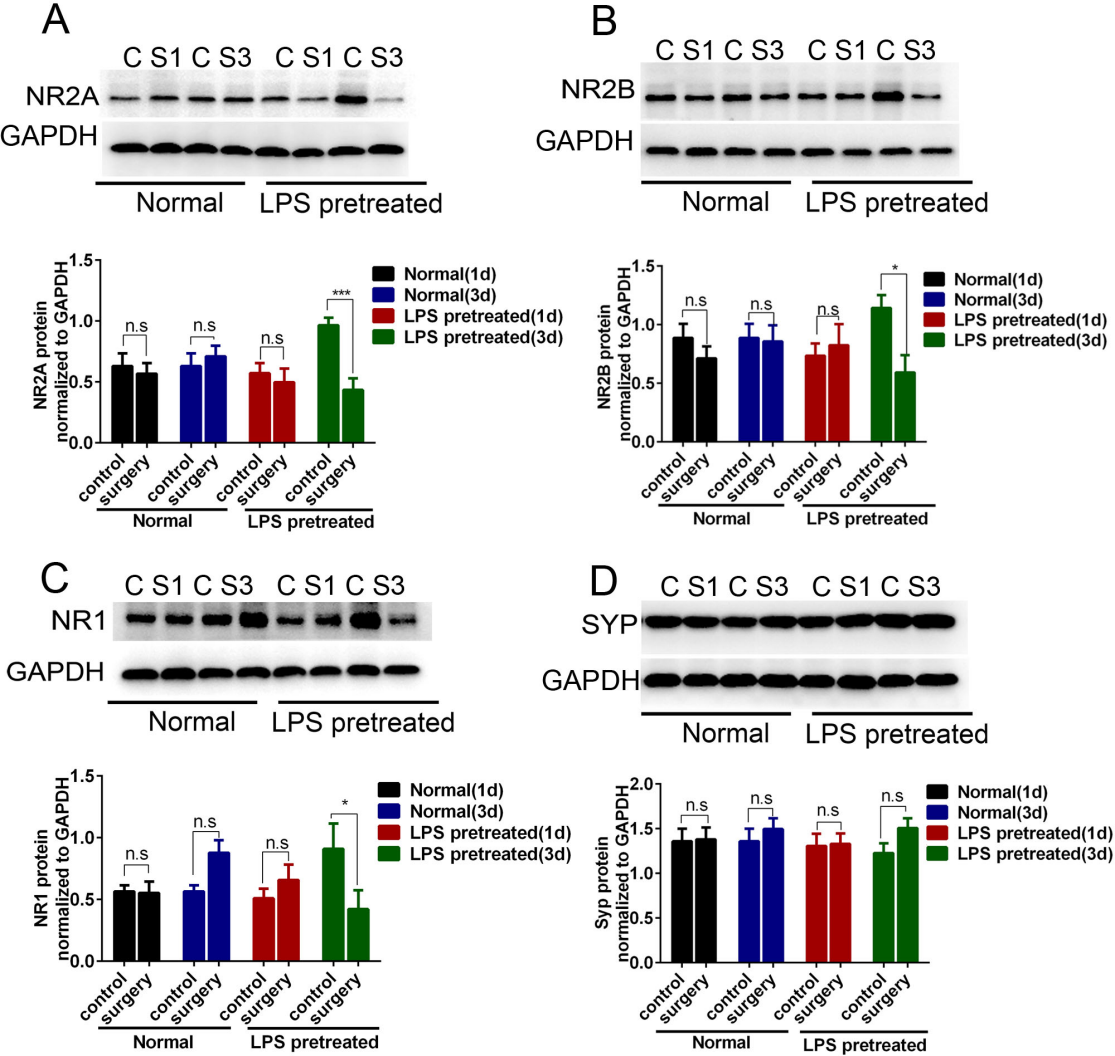
Hepatolobectomy induced the selective loss of NMDA receptors in the hippocampus of LPS pretreated mice, but not in age-matched normal adult mice. Hippocampal synaptic changes analyzed by western blotting. (A-C) Hepatolobectomy reduced the levels of NR2A, NR2B, NR1 in the hippocampus of LPS pretreated mice on day 3. LPS pretreated mice vs. LPS pretreated mice + surgery, p = 0.004 for NR2A, p = 0.03 for NR2B, p = 0.02 for NR1 (D) No significant difference in SYP expression was observed across the groups, p>0.05. LPS pretreated mice (1d) correspond to 6 days survivors, LPS pretreated mice (3d) correspond to 8 days survivors. All data were presented as mean±SEM and analyzed by two-way ANOVA; n = 4 in control part, n = 3 in surgery part; *p<0.05, **p<0.01.

Synaptic plasticity is the structural base of cognitive changes[39]. The subunits NR2A, NR2B and NR1 of NMDA receptors and vesicle protein synaptophysin are closely involved in the synaptic plasticity[40]. Hence we measured the effects of surgery on these protein. As observed in Fig 4, hepatolobectomy obviously decreased the hippocampal expression of NR2A, NR2B, NR1 in LPS pretreated mice on the third day after surgery (p = 0.004 for NR2A, p = 0.03 for NR2B, p = 0.02 for NR1), but not on the first day after surgery(p>0.05 respectively, Fig 4A–4C). However, no obvious difference was detected in the expression of synaptophysin (p>0.05, Fig 4D). In contrast, the expression of those protein in the hippocampus of normal mice 1d and 3d after surgery were similar to that of the control(p>0.05 respectively, Fig 4A–4C).

### Hepatolobectomy obviously increased ROS production in the hippocampus of LPS pretreated mice, but not in aged-match normal adult mice

**Fig 5:**
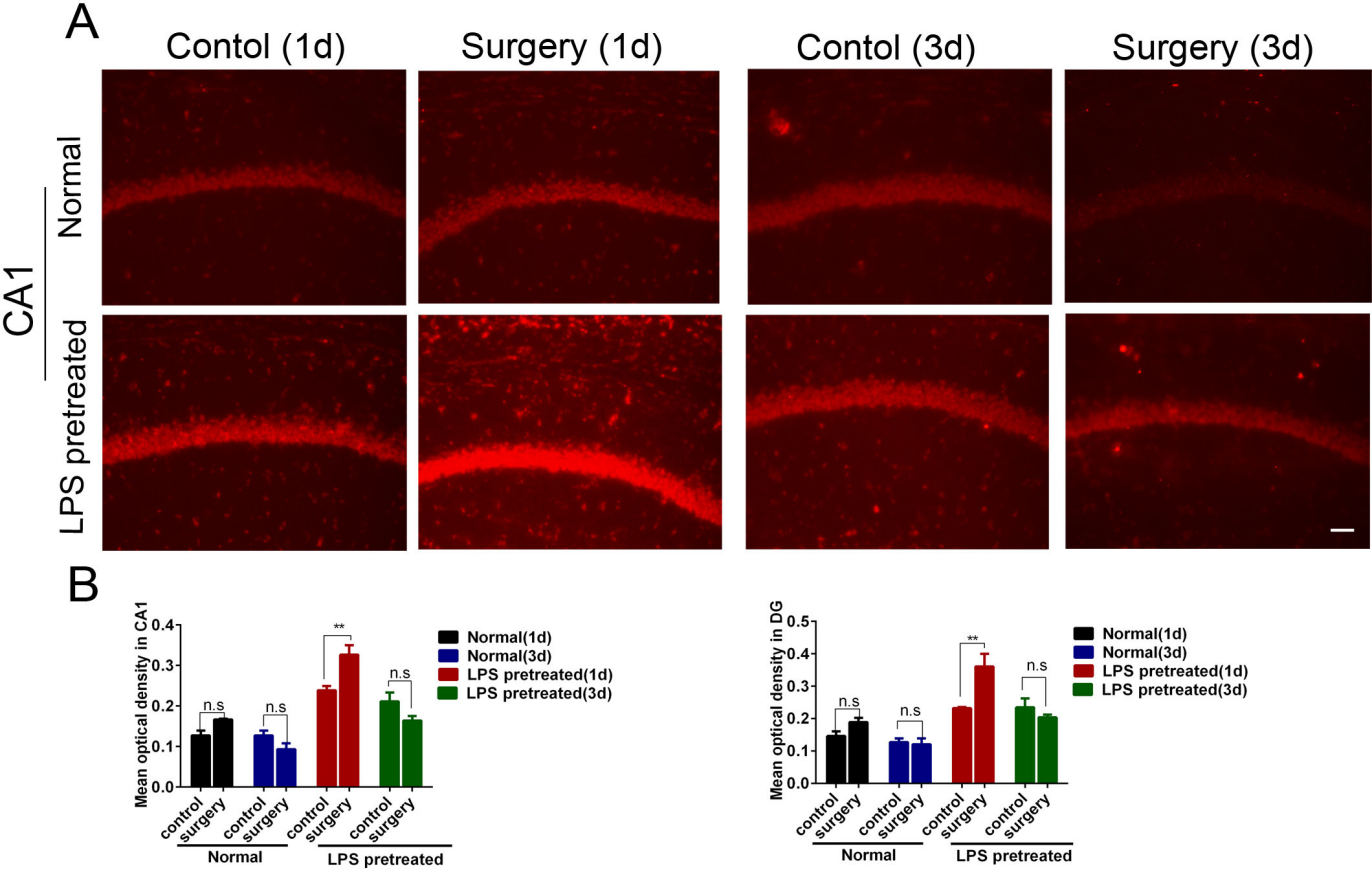
Hepatolobectomy obviously increased ROS production in the hippocampus of LPS pretreated mice, but not in age-matched normal adult mice. (A)Representative images captured from hippocampal CA1. (B) Densitometric quantification of Et fluorescent signal showed higher level of ROS in LPS pretreated mice 1d after surgery. LPS pretreated mice (1d) correspond to 6 days LPS pretreated survivors, LPS pretreated mice (3d) correspond to 8 days LPS pretreated survivors. All data were presented as mean±SEM and analyzed by two-way ANOVA; n = 3 per group; **p<0.01. Bar = 50μm.

It’s reported that oxidative stress may be involved in the occurrence of POCD[9,10]. We used ROS production to measure the level of oxidative stress in the hippocampus. As showed in Fig 5, the LPS pretreated surgery mice exhibited increased ROS generation in the hippocampus as compared to that of the control condition 1d after hepatolobectomy (p = 0.002 in CA1, p = 0.001 in DG, Fig 5A and 5B), while the normal mice didn’t.

## Discussion

Accumulating evidences have increased people's understanding of POCD. Age, surgery’s type, preoperative cognitive level, surgery’s duration and postoperative infection were the main risk factors of POCD [4,17,41,42]. Neuroinflammation[6,7,8], NMDA receptor subunit loss[12], and oxidative stress[9,10] all contribute to the development of POCD. However, it is unknown why elderly patients of the same age and undergone same surgeries have different incidence to develop POCD. In order to answer this question, we tried to construct a model with pre-existing weakness at first. Previous researches reported that patients who suffer from critical illness such as sepsis exhibit great vulnerability to subsequent disability, mortality and cognitive impairment[43]. Based on this, adult mice received intraperitoneal injection of lipopolysaccharide (LPS, 8mg/kg)[23]. We found that the LPS pretreated mice had greater physical frailty index, higher level of neuroinflammation, and lower neuronal activity, which suggested higher level of individual weakness than the age-matched mice. Furthermore, the LPS pretreated mice were obviously different from the Alzheimer disease transgenic model mice, which didn’t show higher physical frailty index and couldn’t really imitate the elderly mice[15]. On the basis, we further detected the effects of surgery on the postoperative cognitive function of LPS pretreated mice and age-matched adult mice. Surgery induced obvious impairments in executive function, learning and memory in LPS pretreated mice, but not in age-matched adult mice. In addition, surgery also induced obvious POCD related pathological changes including exaggerated neuroinflammation, obvious NMDA receptor subunit loss, strong oxidative stress in LPS pretreated mice. In contrast, these pathological changes didn’t occur in age-matched adult mice after surgery. These results are consistent with our previous study. Surgery induced obvious POCD, heightened neuroinflammation, and synapse loss in aged rats, but not in adult rats[11]. These data suggested that increased pre-existing individual weakness is critical for the occurrence of POCD in mice of the same age undergone the same type of surgery.

Previous studies have shown that patients who exhibited preoperative cognitive impairment, reduced hippocampal volume or decreased blood flow in the left middle cerebral artery have a higher incidence rate of POCD compared with the age and procedure matched patients with no preoperative co-morbidities[17,18,19], which suggested the important role of brain vulnerability in modulating POCD’s occurrence and development. In addition, previous studies also showed the important role of surgery-induced peripheral inflammation in POCD’s occurrence and development[44]. Furthermore, surgery-induced peripheral inflammation is obvious higher in elderly individuals than the younger counterparts[45]. In our study, LPS pretreated mice have both higher body frailty index and brain vulnerability than the aged-match mice, thus it is still unknown which is more important in the occurrence of POCD in mice of the same age.

In the study of Fidalgo AR[46], 50ng/kg Lipopolysaccharides(LPS) was added prior to surgery to increase peripheral immune response and investigated the important role of peripheral inflammation in POCD. It is obviously different from the aims of our study. In our study, 8 mg/kg LPS was used 5 days before surgery to increase preoperative individual weakness of the mice. In addition, some limitations of our study should be pointed out. 1) We didn’t investigate the effect of LPS on the other areas of the brain except hippocampus. The reason why we chose hippocampus for mechanism exploration is that hippocampus is closely involved in surgery-induced cognitive impairment[16]. 2) We still don’t know whether the postoperative loss of NMDA receptor subunits decreases the synaptic plasticity or glutamate excitotoxicity, although we detected the loss of NMDA receptor subunits in LPS pretreated mice after surgery. 3) there were no mice only received anesthesia in our study, it is not clear if the POCD is caused by anesthesia or surgery.
